# Farmers’ Biosecurity Awareness in Small-Scale Alpine Dairy Farms and the Crucial Role of Veterinarians

**DOI:** 10.3390/ani14142032

**Published:** 2024-07-10

**Authors:** Marica Toson, Manuela Dalla Pozza, Piera Ceschi

**Affiliations:** 1 Istituto Zooprofilattico Sperimentale delle Venezie, 35020 Padova, Italy; 2 Department of Prevention—Hygiene and Public Health Service, South Tyrol Health Service, 39042 Bressanone/Brixen, Italy

**Keywords:** biosecurity, mountain farming, disease prevention, AMR, vaccination

## Abstract

**Simple Summary:**

Biosecurity measures are necessary to the development of a family alpine farming system. In this study, good practices and areas of improvement have been investigated. In particular, practices related to quarantine, cleaning, waste milk feeding to calves, immunization, and diagnostics could be improved to implement optimal biosecurity measures. Farmers confirm that they are willing to adopt effective preventive measures under the recommendation of the veterinarian, and this attitude may be crucial to designing multisectoral participatory activities and strategies. Further investigations and the development of customized applications and digital toolkits are now required to better understand farmers’ drivers toward biosecurity and to provide support.

**Abstract:**

Background: Biosecurity measures are essential to improve animal health and welfare, tackle antimicrobial resistance (AMR), minimize the burden of infectious diseases, and enhance the safety, security, and quality of sustainable agricultural products. However, the implementation of these measures in small mountain farms can be influenced by several factors, which probably differ from the main variables that affect intensive farming systems. Methods: This study investigated the awareness among farmers regarding the implementation of biosecurity measures at the local level in small dairy farms located in the Autonomous Province of Bolzano/Bozen (Italy). In order to determine to what extent these measures are in line with the recommendations, a questionnaire was conceptualized and sent by post to a representative group of local farmers. The initiative was voluntary and anonymous. Results: A total of 50 farmers responded to the questionnaire, resulting in a response rate of 27.5%. This study confirms that most of the farmers are familiar with biosecurity, and 38% of them know its comprehensive definition. The results indicate that 77% of respondents are willing to implement preventive measures to reduce the use of antimicrobials, and 76% of them acknowledge that they would follow the recommendations provided by veterinarians. In agreement with other studies, the role of the veterinarian as a person of trust among farmers is confirmed. Conclusions: Main strengths and areas of improvement have been identified. Additional data and effective tools are needed to better investigate drivers toward biosecurity and to implement impactful and practical measures for mountain farming.

## 1. Introduction

Biosecurity is defined by the European Regulation 2016/429 (“Animal Health Law”) as “the sum of management and physical measures designed to reduce the risk of the introduction, development, and spread of diseases to, from, and within an animal population or an establishment, zone, compartment, means of transport, or any other facilities, premises, or location” [1].

This definition emphasizes the importance of prevention tools to keep infection pressure as low as possible in order to protect animal health and welfare. Importantly, implementing stronger biosecurity on dairy farms also has an impact on food safety, food security, and public and even environmental health [2]. 

The implementation of biosecurity measures has been strengthened in the last decade at the national level in Italy, and the development of strategies for prevention of animal infectious diseases represents one of the seven pillars of the National Action Plan on Antimicrobial Resistance (Piano di Contrasto all’Antimicrobico Resistenza—PNCAR), adopted for the first time in 2017 [3]. Antimicrobial resistance (AMR) is a major public health concern [4], and the association between biosecurity and antimicrobial use in farmed animals is the subject of intense research [5]. To translate the principles of the National Plan into action at the local level, it is necessary to implement appropriate measures and good practices to prevent diseases that otherwise have to be treated with antimicrobials. For this reason, local stakeholders’ awareness about biosecurity and potential areas for improvement needs to be addressed. 

During the last three years, the Directorate General of Animal Health and Veterinary Medicines of the Italian Ministry of Health has implemented an innovative information system, named ClassyFarm, which allows farms to be categorized according to risk. It is available to official veterinarians, farm veterinarians, and farmers, and it allows the processing of data related to the following assessment areas, i.e., biosafety, animal welfare, health and production parameters, animal feeding, antimicrobial consumption, and lesions found at slaughterhouses [6]. However, in 2023, an accurate investigation of Classyfarm’s implementation in the Autonomous Province of Bolzano/Bozen was published, and the authors raised concerns about the appropriateness of the questionnaires developed, as the peculiarities of small mountain dairy farms are not taken into consideration [7]. 

In accordance with paragraph 43 of the European “Animal Health Law”, measures need to be applied with a certain degree of flexibility. In particular, specific productions, species or categories of animals, and local circumstances must be considered. Therefore, a study about biosecurity was designed, focusing on specific variables that may have an impact on the implementation of these measures in alpine dairy farms. 

In order to not only collect specific additional data about this farm system but also to highlight strengths in disease prevention and to provide practical evidence-based recommendations to local farmers, if required, a questionnaire consisting of 74 questions related to farm characteristics, general breeding management, selling and purchasing policy, management of calves, management of adult cattle, and relevant opinions was conceptualized. A representative subgroup of farms was selected, and questionnaires were sent by post. Enrollment into the initiative was voluntary and anonymous.

## 2. Materials and Methods

### 2.1. Study Area and Selection of the Farms

The area of the Autonomous Province of Bolzano/Bozen covers 7400 square kilometers; it has 535,774 inhabitants according to the 2021 census [8], and it is entirely located in the Alps. 

This province is divided into four main local health districts, which are subdivided into 20 subdistricts. The district of Bressanone/Brixen was chosen to perform this study. This area includes three subdistricts, i.e., Alta Valle Isarco/Wipptal (with 5 municipalities), Bressanone/Brixen (with 6 municipalities), and Chiusa/Klausen (with 6 municipalities). In this province there are three official languages, i.e., German, Italian, and Ladin. 

According to the last agricultural census [8], the total number of cattle registered in the province was 132,784 and the number of registered farms was 8315. The median herd size was 15 animals per dairy farm. 

A sample of 800 dairy farms was selected from a total of 3285 dairy farms located in the province of Bolzano/Bozen that were registered in the national animal register. Then, from this sample, all the farms located in the district of Bressanone/Brixen were included in the investigation, and, finally, a sample of 182 farms was randomly selected in order to achieve an assumed 25% response rate (with at least 44 expected respondents), based on the existing literature [9]. The selection of farms involved simple random sampling performed through the SAS procedure (PROC SURVEYSELECT PROCEDURE of SAS 9.4 software (Copyright @ 2002–2012 by SAS Institute Inc., Cary, NC, USA), stratified by municipality, and categorization of the number of animals (on the basis of median animal capacity: median = 15 animals).

### 2.2. Questionnaire Development, Distribution, and Collection 

A draft questionnaire was developed in 2021, and four farmers, two veterinary practitioners, and an expert in mountain agriculture and milk production tested it and provided feedback. 

To facilitate its completion and the subsequent analyses, the questionnaire was divided into 6 main sections as follows: general information (*n* = 25 questions), general breeding management (*n* = 6 questions), selling and purchasing of animals (*n* = 9 questions), management of calves (*n* = 8 questions), management of adult cattle (*n* = 5 questions), and opinions (*n* = 21 questions). The questions referred to the sanitary situation in the last 12 months. Both fill-in-the-blank and multiple-choice questions were presented to the farmers. A 5-point Likert scale was used to design the last part of the questionnaire to assess farmers’ opinions towards biosecurity. 

Questionnaires were sent by post at the beginning of February 2022, and farmers had two months to return them to their local veterinary service. As the aim of the initiative was neither to assess the compliance of the farmers’ behaviors and practices with the legislation in force nor to provide individual counseling, participation was voluntary and anonymous. An invitation letter was added to the questionnaire to inform the farmers about the initiative. The questionnaire was written in German.

### 2.3. Data Analysis (Statistical Methods, Multivariable Models, and Associations) 

Data collected through the paper survey were stored and validated in an Excel file. Validation has been performed through a consistency check of the answers reported for different variables and questions but logically related. Descriptive statistics were applied to the data. The association between categorical variables was evaluated through Pearson’s chi-squared test or Fisher’s exact test. Data were analyzed using STATA 17.0, and results with a *p*-value < 0.05 were considered significant.

An ordered regression model, as conducted by Robichaud et al., 2019 [10], was performed to assess the variables that were most closely associated with the rank of agreement with some items. However, the marginal analysis did not show any significant associated variables to put into the multivariable ordinal model, even if we considered a 5-point Likert scale or a 3-point Likert scale, created by grouping the extreme values. The Likert data have been presented as categorical data through histogram. 

## 3. Results

### 3.1. General Data 

**Figure 1:**
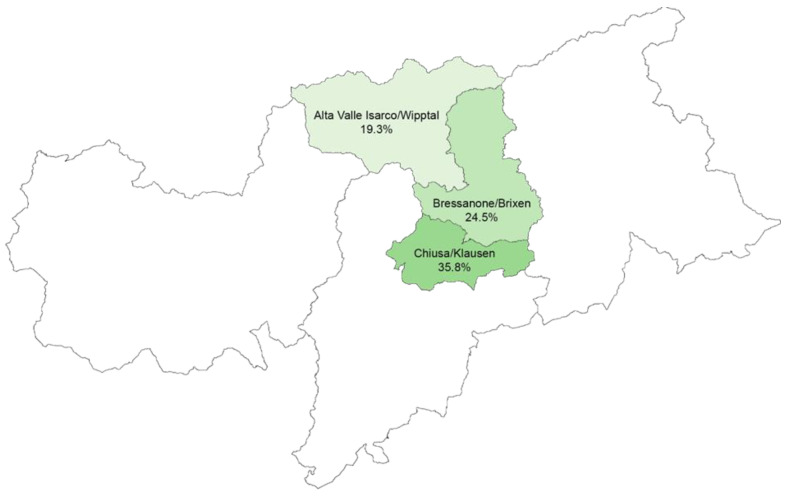
Area of study and geographical distribution of the respondents.

A total of 50 farmers filled in and returned the questionnaire to their local Office of Veterinary Services, representing a response rate of 27.5% (Table 1). Questionnaires were returned from 14 of the 17 municipalities included in the study. One farmer omitted to specify the municipality where their farm was located. Figure 1 shows the area of study in the Autonomous Province of Bolzano/Bozen and the geographical distribution of the respondents in the district of interest. 

Three questionnaires were considered not eligible for analysis. Two of them were uncompleted, and one was filled in by a farmer who reported having only two bovines (one cow and one calf), and the milk was kept for domestic use only. Among the respondents, two of them were women younger than 60 years old, 27 of them were men younger than 60 years old, and 15 of them were men over 60 years old. Two respondents did not answer these questions. Data are displayed in Table 2. 

Most of the farmers (61.7%) reported working full-time on-farm, and almost one-third of them (28%) offered on-farm holiday accommodation. Overall, 83% of the farms were located in the countryside, and 66% of the farms were located above 1000 m above sea level (masl). No significant association (chi-squared test: *p*-value = 0.335) was found between the husbandry system (tie- vs. free-stall) and the location of the farm (in terms of sea level and urban or rural areas). 

The median size of the herd was 15 cows per farm; 62% of the respondents had more than 15 cattle (26.8 animals on average), 36% had fewer, and 2% omitted to provide this information. 

Almost two-thirds of the farms (60%) produced conventional milk, while 26% adhered to specific production systems (organic, hay milk, and organic hay milk). Among the farms, 70% sold milk exclusively to a dairy plant, while 13% also sold small quantities of milk and milk products to end consumers (2% unknown). Overall, 15% of the farmers did not sell milk during the period of study (dry period). Data are shown in Table 2. 

### 3.2. Health Literacy 

One question about the definition of biosecurity was included in the questionnaire. The correct answer included both measures in place to avoid the spread of pathogens within the farm (internal measures) and to prevent pathogens from entering the farms (external measures). Overall, 38% of the farmers identified the comprehensive definition of biosecurity (including both internal and external measures); 15% of the respondents associated the meaning of this concept with either the internal or the external biosecurity definition; 9% indicated that it relates only to organic farms; and 38% did not know the definition. 

### 3.3. Breeding Management 

At least 68% of the farmers reported that they kept cattle in tie-stalls. Most of the farmers (78%) separated animals by age and/or production group. Both artificial insemination (83%) and natural breeding (17%) were practiced. 

### 3.4. Diseases 

A positive association between herd size and the presence of reported diseases was observed (Kruskal–Wallis test: *p*-value = 0.0025 for mastitis; *p*-value = 0.0246 for diarrhea); in fact, the median number of animals was higher in those farms that experienced cases of mastitis or diarrhea (median number of animals: 22 vs. 9 for mastitis; 20 vs. 8 for diarrhea). Whereas no significant association between the husbandry system and mastitis was found (*p*-value = 0.428), there was a significant association between the free-stall system and the presence of diarrhea (*p*-value = 0.002). 

### 3.5. Working Organization and Cooperations 

**Figure 2:**
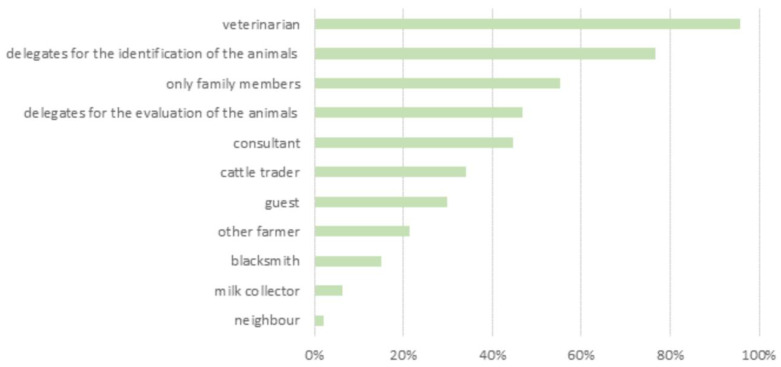
People allowed to enter the stables.

**Figure 3:**
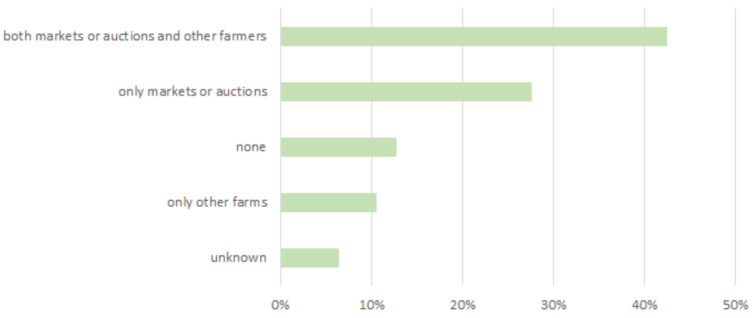
Attendance of facilities where animals are kept.

Overall, 6% of the farmers reported the presence of employees on their farm. Also, cooperation with auxiliary forces during the peak summer mowing season was reported. These people are usually recruited on a voluntary basis, not paid, and not allowed entry to the stables where animals are kept. More than 50% of the farmers stated that only family members were allowed to enter the stables where animals are kept, with some exceptions (veterinarians, authorities, etc.) (Figure 2). Stable entry is usually allowed for veterinarians (96%), followed by delegates of the local farmers’ association, people in charge of the identification of animals (77%), and the evaluation of animals (47%), respectively (Figure 2). Most of the respondents reported regularly attending animal trade or exhibition fairs (70%) and stables belonging to other farms (53%). The practice of tool and equipment sharing was adopted by 21% of the respondents. Overall, 42% of farmers attended both markets/auctions and other farms, while 13% of them followed more restrictive behavior (Figure 3). 

### 3.6. Maintenance and Cleaning Procedures 

Milking equipment and installation maintenance occurred regularly in 51% of the farms, while 30% of the farmers indicated that service operations took place every 3 to 5 years or as required. Overall, 19% did not answer this question, and, among them, 15% declared keeping non-lactating cows. Meanwhile, 42% of the respondents indicated that they clean drinking troughs when they are dirty. 

Around 60% of the farmers complied with regular preventive hygiene practices (cleaning of the vehicles used for animal transport, of water troughs, and calf shelters). No statistically significant differences between full- and part-time farmers were detected. 

### 3.7. Other Potential Risk Factors 

Dead animals are removed from the farm by a specialized company in 62% of cases, while 38% of the farmers reported using their own vehicles to transport carcasses to the local disposal plants. 

Manure management is usually mechanical (66%). Only 2% of the farmers use manure for biogas production. 

Natural water spring access is allowed to cattle in 62% of farms. 

Overall, 64% of the farmers confirmed that only cattle were kept on their farms. However, 27% of them cannot exclude contact with other animals (both domesticated and wild). At least 91% of respondents acknowledged that animals are kept in summer pastures, where the presence of other species (pets, wild and farmed animals) cannot be ruled out. 

**Figure 4:**
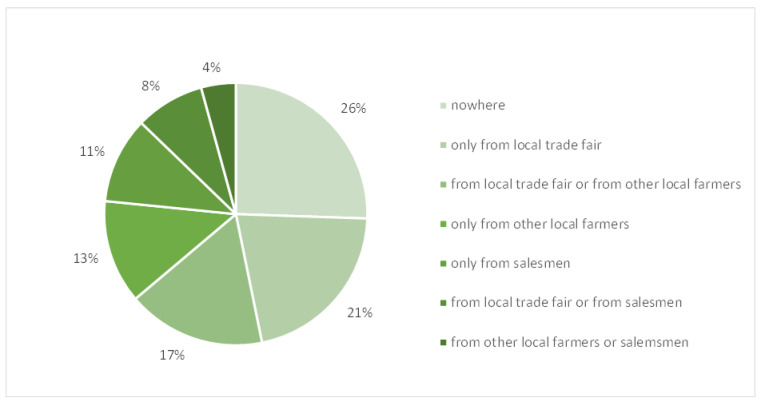
Purchase of cattle (private, local trade, etc.).

More than 60% of the farmers reported selling cattle only if necessary, and, when they sell them, they usually contact professional salesmen (89%). The majority of farmers usually purchased cattle at the local trade fair, from other local farmers or from salesmen (Figure 4). Moreover, 4% of farmers (N = 2) reported applying a quarantine period after purchasing animals. Farmers report that they do not place animals in quarantine, either because it is not possible (21%), or because it is not considered to be necessary (55%). Usually, cattle are purchased inside the province (55%), from abroad (Austria and Germany), or the neighboring Autonomous Province of Trento.

### 3.8. Calf Management

Calf shelters are usually cleaned only if necessary (38%). Most of the farmers avoid feeding with milk of cows treated with antibiotics during the withdrawal period (60%), while some of them feed with mastitic milk (45%). During the last 12 months, 43% reported administering antibiotic treatments, and 12% reported administering vaccinations. Regarding diseases, in 23% of cases, diagnostic testing was performed. 

### 3.9. Cattle Management 

During the last 12 months, 68% of the farmers reported administering antibiotic treatments, and 4% reported administering vaccinations. Regarding disease, in 17% of cases, diagnostic testing was performed, and 8% of the farmers used antimicrobial susceptibility testing. Overall, 51% of the farmers reported fewer than five cases per year of mastitis, and 21% reported no cases at all. Overall, 26% of farmers did not answer this question. 

### 3.10. Opinions 

**Figure 5:**
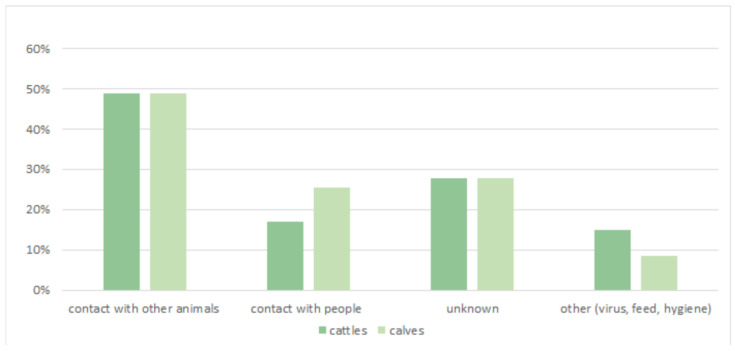
Factors that are considered to be relevant in the transmission and spread of infectious diseases at farm level.

Overall, 53% of the farmers agreed that their adult cattle may be at risk of infectious diseases, while 34% of them were confident that they were not. Diarrhea in calves (89%) and mastitis in cows (43%) are considered to be the most relevant diseases. Meanwhile, 49% of the respondents were confident that contact with other animals represents the most relevant risk factor, followed by unknown variables (28%), contact with people, feed, the purchasing of cattle, viral agents, and poor hygienic conditions (Figure 5). 

As regards biosecurity, the most effective actions taken to prevent infectious disease were the cleaning of clothes and boots, preventive measures, and the isolation of sick animals. Farmers who do not take any action explained that these measures are too expensive, not applicable, or even not necessary (as animals are healthy). In 28% of these cases, the farmers asserted that they did not know what more they should do. 

The main drivers of implementing preventive measures were to minimize the use of antimicrobials (74%), and to save money (68%). 

**Figure 6:**
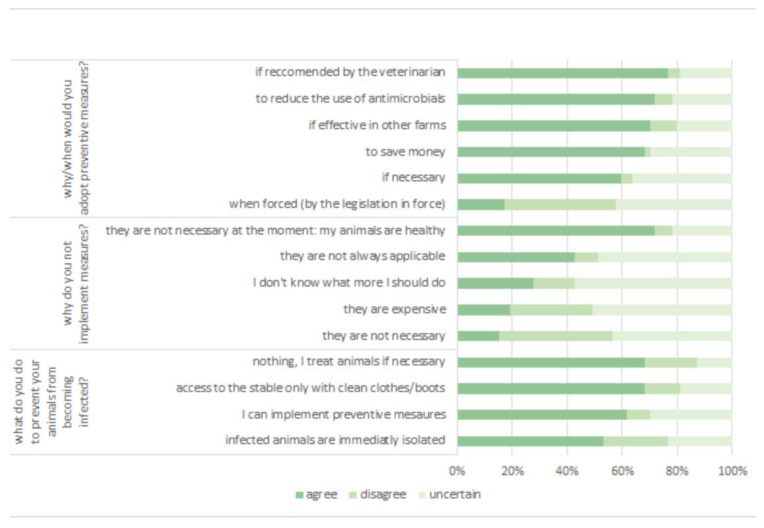
Opinions about biosecurity.

Some farmers stated that they will adopt preventive measures only if they are effective on other farms (60%), if necessary (59%), or when forced (17%). Overall, 77% of them were willing to adopt additional measures under the recommendations of a veterinarian. Moreover, 62% of them confirmed that a veterinarian had already provided information about biosecurity, and at least 51% showed interest in this topic. Farmers’ answers are displayed in Figure 6. For clarity, data are displayed on a three-point Likerts scale where the group “agree” reports the answers of farmers who strongly agree and agree with the questions, the group “disagree” reports the answers of people who are in strong disagreement and disagreement, and the group “uncertain” represents the respondents who are not sure about the answer.

## 4. Discussion

Due to its position, Italy presents different climatic conditions, varying from Alpine to the Po Valley, Mediterranean, and Peninsular climate. Dairy farming in Italy varies within the country also according to different local settings, traditions, and geographic conditions. In Italy, four main regions located in the northern part of the country deliver 66% of the national milk [11]. These regions are not entirely located in the Alps; most of their farms are in the flatland, and they keep a large number of animals in free stalls all the year. On the contrary, the Autonomous Province of Bolzano/Bozen is entirely located in the Alps, and it is characterized by small dairy farms, where animals are often kept in tie-stalls. However, summer grazing in the Alpine pastures is a typical practice that allows cattle to move outside the farms in better climate conditions and helps reduce the workload for farmers. This practice is also linked to the local traditions and the development of tourism. On the other hand, these characteristics may influence the burden of infectious diseases, their spread, and the measures taken to contrast them. The discussion highlights peculiarities that have been investigated. 

### 4.1. The Context and the Response Rate

The response rate is relevant to assessing the quality of a survey [12]. In this study, 27.5% of the questionnaires were filled in and returned. As expected, this rate is lower in comparison to similar studies that included an additional reminder for selected farmers and/or a visit to the farm, which reported response rates of over 40% [13,14], but higher than expected (25%) when compared to other studies and online surveys [9]. In this study, factors that may have had a positive impact on the response rate include the selection of a well-defined target population (farmers that live in the same district and breed cattle) and the relevance of the topic for this category of people. Also, the choice to avoid contacting the participants in advance and reminding them about the survey probably affected this result. However, it is not possible to ascertain if this had a positive or a negative effect. The authors decided to prioritize the anonymity of the respondents in order to gain insights into the opinions and practices without any possible influence of intermediary persons before or during the completion of the questionnaires. As a higher number of potential participants and the use of incentives do not necessarily guarantee an increase in response rate (11), the study was not extended to other districts and no incentives were offered. 

The debate is still open: whether postal surveys can result in higher or lower response rates compared to e-mail surveys [15]. We preferred to send the survey per post, also assuming that every farmer would receive it, in order to reduce the potential impact of respondents’ attitude towards technology on the non-response bias. 

A low number of farmers were women, and most of the respondents were men over 40 working full-time on farms located outside the villages, above 1000 masl, producing conventional milk. These data provide insights into the demographics of farmer society; however, they are not sufficient to understand the underlying factors that characterize the reality of local dairy farms. Importantly, women play an essential role, making invaluable contributions to livestock, family, and, if present, farm holiday management. Moreover, often more than one generation live together on a farm; youths have a job in the main town, and, in addition, they provide regular support to their parents, who work full-time on their farm. This approach is crucial to allow the existence of a traditional alpine farming system.

### 4.2. Health Literacy and Awareness on Preventive Measures

In accordance with other studies [16], most of the farmers already knew the complete definition of biosecurity or related it to the definition of external or internal biosecurity. Moreover, it is pertinent to mention that, as the German word for organic farm is “biologisch”, the suffix “bio” could have been misleading. 

Among the participants, the majority declared being interested in additional information about biosecurity measures, and this attitude is a valuable strength. As a matter of fact, the improvement of health literacy is extremely relevant in the veterinary and agricultural sectors, as this could lead to improved compliance and outcomes [17]. This study was also designed to provide farmers with relevant information to share with them their strengths and areas for improvement, to raise awareness on this topic and to improve their knowledge. To meet the demands of farmers and to address their willingness to learn more about preventive measures, on the basis of the results of this study, an awareness initiative can be promoted. 

Behaviors and motivators among farmers have been investigated [18,19], and it has been shown that if preventive measures are not perceived as relevant by farmers, they might end up causing a higher risk of the spread of diseases [18,20,21]. The current study found a low willingness to vaccinate calves (12%), while an even lower percentage of farmers (only 4% of the respondents) had immunized their adult cattle during the last 12 months. As determinants such as age, education, and family size have been associated with the willingness to vaccinate cattle [22], it would be worthwhile to assess which factors and drivers may have an impact on this choice. Moreover, in the future, it will be possible to detailed obtain data from the National Electronic Veterinary Recipe Platform. This tool has recently been implemented by the Ministry of Health [23] to monitor the use of vaccinations and, if required, to design and recommend effective immunization strategies to reduce the incidence of relevant vaccine-preventable diseases in cattle, especially those that can be transmitted to humans. 

On the other hand, one of the factors that motivated the participant to this study to improve preventive measures was the possibility to reduce antimicrobial consumption. As vaccination is one of the most cost-effective biosecurity measures to prevent infectious diseases in cattle and, consequently, to reduce the need for antimicrobial treatments [4,5,24], it would be reasonable to inform thoroughly the stakeholders about the benefits of immunization. 

Nevertheless, it must be considered that some vaccinations are not available. Moreover, the adoption of some immunization programs is not allowed or required, as this province has achieved the status of being officially free from several major animal diseases, including bovine tuberculosis, enzootic bovine leukosis (EBL), and bovine brucellosis. Moreover, additional compulsory eradication programs have been adopted for the control of relevant transmittable diseases such as bovine viral diarrhea virus (BVDV) [25] and infectious bovine rhinotracheitis [26]. The high level of the cattle health status in this province was achieved thanks to veterinary strategic decisions that promoted and strengthened effective surveillance, control, and eradication programs in close collaboration with farmers and their local associations. Appropriate policies were implemented to protect both animal and human health and to guarantee the supply of local safe and sustainable products of animal origin for human consumption. Anyway, surveillance programs for early detection of infectious diseases have to be permanently ensured [27]. These aspects are relevant in this province because the alpine farming, with its prevalent traditional, family-based management system, could be vulnerable to the spread of re-emerging diseases, and the successful marketing of products with high biological value proteins could be threatened. On the other hand, this farming system could be intrinsically resilient to threats. In fact, in this study, we observed a significant association between herd size and the presence of reported cases of mastitis and diarrhea, which represent the most relevant diseases that may occur from the participants’ point of view. This can be explained by the fact that, very often, farmers’ entire families put a particular effort into the daily care of a relatively low number of cattle, in conjunction with the awareness among local farmers of the relevance of both diseases. Under these circumstances, if farmers can be trained in disease detection, they will probably be willing to cooperate with the local veterinary authorities to enhance the local warning system for the prompt detection of signs of other relevant infectious diseases.

### 4.3. The Role of the Veterinarians, Strengths, and Areas of Improvement 

In agreement with other studies [2,16,21,28,29,30], veterinarians have been acknowledged to be trustworthy informants, and the decision to implement preventive measures can be strongly influenced by them. In this study, 62% of respondents had already been informed by a veterinarian about these measures, and, importantly, 77% of the respondents reported that they were willing to improve their biosecurity measures under the recommendations of a veterinarian. 

These results can suggest additional research questions for further studies to better investigate, for example, whether veterinarians are consulted on a regular basis or just for unusual cases, as reported by other authors [31], and how veterinarians can support the change in the long run [32]. 

This study allows us to acknowledge good practices and habits that need to be further encouraged and how the veterinarians can play a pivotal role. For instance, stable entry is usually allowed only to family members who have the primary interest of keeping their animals healthy and to staff who is trained and already has comprehensive knowledge about preventive measures (among them, veterinarians, and delegates of the local farmer association). Under these circumstances, the risk of diseases’ transmission can be considered low, provided that hygiene good practices are respected. In fact, the risks linked with the daily attendance of many farms where both healthy and sick animals are kept and the limited time available for changing the clothes and disinfecting the boots cannot be underestimated. 

Other good practices that are already in place and must be further promoted are wearing clean clothes and boots when entering a pen, the early isolation of sick animals, and the allocation of animals by age or production group. 

On the other hand, areas for improvement also emerged. Only approximately half of respondents confirmed that they perform regular inspections and replacement of milking equipment, while less than half of them cleaned drinking troughs and calf shelters regularly. Even though a high number of farmers purchase cattle, only 4% quarantine their cattle, either because it is not possible or because this practice is not considered to be necessary. At this point, it is crucial to emphasize that mountain farming is demanding and that there are restrictions and limits. In particular, it can be challenging or even impossible to renovate buildings to provide space for quarantine pens or to convert tie-stalls into animal-friendly free-stalls equipped with quarantine boxes. Nevertheless, in this study, it was not possible to find an association between the lack of quarantine practices and the incidence of diseases due to the low number of farmers that do apply quarantine. Interestingly, in accordance with other studies [33], we did not find any association between the tie-stall system and a higher incidence of diseases. On the contrary, a positive association between the free-stall system and the presence of diarrhea was confirmed. 

During the 12 months before this study, 43% of farmers reported the use of antimicrobials. However, diagnostic practices are not common; in fact, only 8% of the farmers indicated the use of antimicrobial susceptibility tests. Moreover, 45% of them reported feeding calves with mastitic milk and 40% with milk of cows treated with antimicrobials during the withdrawal period, even though this practice should be avoided as it is detrimental to calves’ health [34]. Of course, shifting from a curative approach to a preventive strategy requires time and training. However, if farmers and veterinarians agree on shared objectives and perceive counseling as an opportunity, it will be possible to improve both diagnostic and feeding practices [2,13]. 

This study shows that the majority of the farmers purchase cattle at trade fairs from other farmers and salesmen. Even though animals’ movement must comply with the applicable law and, usually, animals are moved from areas and countries with the same health status as this province, purchasing cattle can be a risk in terms of the introduction of diseases into a farm, especially those that are not targets of official control programs. Several factors, such as the frequency and number of animal purchases but also transport practices, play a crucial role. For these reasons, strict hygiene measures at purchase and during transport must be ensured. In particular, salespersons and farmers who regularly attend cattle markets must be aware of potential risks and be trained in preventive measures to minimize the risk of disease spread. 

Other potential factors that need to be monitored are the use of artificial insemination (AI), the sharing of equipment, carcass removal, and manure management. We found that reproduction strategies are mainly the responsibility of veterinarians or trained staff, so this practice should not pose a significant risk. Equipment sharing is not very common; however, the situation needs to be monitored, as it may change in the future due to the need to split the costs of buying and maintaining machines and instruments. Carcasses can either be removed and disposed of by specialized companies or transported by the farmer to a disposal plant. Considering the small herd sizes and the location of the farms (most of them located outside of villages), disinfection baths and gates at farm entrances are not common. As vehicles usually have to enter the premises of several farms, either when they collect milk or when they remove carcasses, truck cleaning and disinfection at the available vehicle washing facilities before leaving them is essential in order to minimize risks. Another peculiarity that must be considered for small alpine farms is the truck traffic, which is not always possible as streets can be too narrow. For this reason, many farmers bring milk tanks directly to local collection points, and this can be considered a useful practice to reduce the daily entry of vehicles into the premises. 

Summer pastures characterize alpine farming, and as expected, we found that a high percentage of farmers (91%) adopt this practice. This means that cattle may have contact with other animals (pets, wild and farmed animals) and access water springs. Even if local farmers identify the variable “contact with animals” as the most relevant risk factor for disease transmission, unfortunately, they lack control over this factor, as animals in pastures are usually kept outdoors and are not supervised all the time. 

The transmission of waterborne diseases represents a specific topic that goes behind the scope of this study. However, we asked farmers two questions about cattle water intake. As potable water is available in local stables, the first question was whether drinking troughs are cleaned, and in 42% of the farms this happens only if they are dirty. The second question addressed the potential access to natural water springs, i.e., as 91% of the farmers bring their cattle to summer pastures, this possibility cannot be ruled out. In the first case, cattle are exposed to risk, and this can be minimized by adopting good cleaning practices. On the other hand, the second scenario may have implications from a public health perspective, as water can be contaminated with pathogens from cattle feces [35]. However, this risk should be negligible, as access to natural water springs is limited by fences. 

### 4.4. Perspectives

In this study, the majority of the farmers showed interest in biosecurity (51%), and they confirmed that they would be willing to adopt preventive measures if they are effective on other farms (60%) and if necessary (59%), in order to reduce the use of antibiotics (74%), and to save money (68%). However, it should be further investigated as to which preventive measures are not applicable, why some respondents prefer to wait and treat animals if necessary, and how farmers can be better supported, as some of them stated that they do not know what more they should do. A high number of farmers said that they do not apply biosecurity measures because their animals are healthy. The value of some good practices that are already in place (cleaning of boots, separation of animals, stall entry allowed only to a restricted number of persons) should be acknowledged, and the intervention of a veterinarian would be crucial to explain why some measures are crucial and should be maintained. 

Mountain farming can be considered as a model for local sustainable development, even if challenges have to be taken into account [36,37,38]. To avoid the risk of decline and abandonment, local farmers have been able to diversify their activities, and they can generate revenue from tourism, which is of vital importance for small-scale farming [39,40,41]. Even though a low number of women and farmers under 40 responded to the questionnaire, this does not necessarily mean that women and young people are not involved in working activities on farms. Unless proven otherwise, we suggest that women can feel empowered and help to develop touristic activities on farms, thus contributing to farmers’ families’ income. Furthermore, mountain agriculture may be attractive for young people who can adapt innovative concepts to ensure the modernization and sustainability of this sector [42,43].

This study highlights the importance of drivers (such as a reduction in the use of antimicrobials and cost savings) and of the main actors (in particular veterinarians), who may play a crucial role in the implementation of preventive measures. 

Prevention measures are associated both with costs (for example, vaccinations and testing of animals) and benefits (healthier animals are more productive). In this study we included two questions, one related to the willingness to save money in the long term as a driver to improve biosecurity, and one to better understand whether these measures are considered too expensive. Most of the farmers confirm that improving biosecurity brings more benefits in terms of saving money, and less than 20% of the respondents consider these measures expensive. These findings confirm an optimistic attitude toward biosecurity. As interest in this topic is growing and promising projects about biosecurity’s cost-effectiveness are being promoted at the international level, in the future it will be possible to provide evidence-based practical guidance also from this perspective.

As the enrollment was voluntary and anonymous, no farm interviews or reminder calls were planned. Because of this, it was not possible to address specific answers to better investigate some factors that could influence the outcomes or ask for clarifications for some missing or unclear answers. As studies on farmers’ perspectives, attitudes, beliefs, and motivations regarding biosecurity implementation are effective in order to understand why some measures are not implemented and to optimize the undertaking of good practices [44,45], further specific surveys focusing on behavioral changes are recommended. In addition, as the role of self-evaluation tools like *Classyfarm* [6] and Biocheck^®^ [19] has been consolidated in improving the awareness of biosecurity and strengthening the implementation of specific preventive measures, we would recommend the development of additional applications and digital toolkits for self-evaluations customized to small-scale family alpine farms.

## 5. Conclusions

This study highlights several strengths of small-scale alpine dairy farms and those practices related to quarantine, cleaning, waste milk feeding to calves, immunization and diagnostics that could be improved to implement optimal biosecurity measures. As farmers confirmed that they were willing to adopt effective preventive measures under the recommendations of a veterinarian, this attitude will be crucial to designing multisectoral participative initiatives. Further investigations and the development of customized applications and digital toolkits are now required to better understand local farmers’ drivers toward biosecurity and to provide support.

## Figures and Tables

**Table 1:** Total respondents (N = 50).

Geographical Distribution of Participants			
Sub-Districts	No of Selected Farms	No of Respondent Farms	Respondents/Selected
Alta Valle Isarco/Wipptal	62	12	19.3%
Bressanone/Brixen	53	13	24.5%
Chiusa/Klausen	67	24	35.8%
Unknown		1	-
**Total**	**182**	**50**	**27.5%**

**Table 2:** General information (questionnaires included in the analysis, N = 47).

Variable	No	% ^1^
Groups (Age and Gender)		
Women < 60 years old	2	4.3%
Men < 60 years old	27	57.4%
Men > 60 years old	15	31.9%
Unknown	3	6.4%
**On-farm Employment**		
Full-time	29	61.7%
Part-time	17	36.2%
Unknown	1	2.1%
**Farm type**		
Holiday accommodation	13	27.7%
Private	34	72.3%
**Location**		
Urban area	7	14.9%
Countryside	39	83.0%
Unknown	1	2.1%
**Altitude**		
Above 1000 masl	32	68.1%
Under 1000 masl	15	31.9%
**Milk Production**		
Conventional milk	28	59.6%
Organic, hay milk, organic hay milk	12	25.5%
Only dry cows (at the moment)	7	14.9%
**Production and Delivery**		
Dairy plant	33	70.2%
Dairy plant, small quantities of milk, and milk products to end consumers	6	12.8%
Only dry cows (at the moment)	7	14.9%
Unknown	1	2.1%

^1^ Sum of the categories within a variable and within a column equals 100%.

## Data Availability

Relevant questions are reported in the manuscript. The full text questionnaire (in German) is available upon request to interested researchers.
